# Scalable Iterative Classification for Sanitizing Large-Scale Datasets

**DOI:** 10.1109/TKDE.2016.2628180

**Published:** 2016-11-11

**Authors:** Bo Li, Yevgeniy Vorobeychik, Muqun Li, Bradley Malin

**Affiliations:** Vanderbilt University

**Keywords:** Privacy preserving, weak structured data sanitization, game theory

## Abstract

Cheap ubiquitous computing enables the collection of massive amounts of personal data in a wide variety of domains. Many organizations aim to share such data while obscuring features that could disclose personally identifiable information. Much of this data exhibits weak structure (e.g., text), such that machine learning approaches have been developed to detect and remove identifiers from it. While learning is never perfect, and relying on such approaches to sanitize data can leak sensitive information, a small risk is often acceptable. Our goal is to balance the value of published data and the risk of an adversary discovering leaked identifiers. We model data sanitization as a game between 1) a publisher who chooses a set of classifiers to apply to data and publishes only instances predicted as non-sensitive and 2) an attacker who combines machine learning and manual inspection to uncover leaked identifying information. We introduce a fast iterative greedy algorithm for the publisher that ensures a low utility for a resource-limited adversary. Moreover, using five text data sets we illustrate that our algorithm leaves virtually no automatically identifiable sensitive instances for a state-of-the-art learning algorithm, while sharing over 93% of the original data, and completes after at most 5 iterations.

## 1 Introduction

VAst quantities of personal data are now collected in a wide variety of domains, including personal health records, emails, court documents, and the Web [1]. It is anticipated that such data can enable significant improvements in the quality of services provided to individuals and facilitate new discoveries for society. At the same time, the data collected is often sensitive, and regulations, such as the Privacy Rule of the Health Insurance Portability and Accountability Act of 1996 (when disclosing medical records) [2], Federal Rules of Civil Procedure (when disclosing court records) [3], and the European Data Protection Directive [4] often recommend the removal of identifying information. To accomplish such goals, the past several decades have brought forth the development of numerous data protection models [5]. These models invoke various principles, such as hiding individuals in a crowd (e.g., *k*-anonymity [6]) or perturbing values to ensure that little can be inferred about an individual even with arbitrary side information (e.g., *∊*-differential privacy [7]). All of these approaches are predicated on the assumption that the publisher of the data knows where the identifiers are from the outset. More specifically, they assume the data has an explicit representation, such as a relational form [8], where the data has at most a small set of values per feature [9], [10], [11], [12].

However, it is increasingly the case that the data we generate lacks a formal relational or explicitly structured representation. A clear example of this phenomenon is the substantial quantity of natural language text which is created in the clinical notes in medical records [13]. To protect such data, there has been a significant amount of research into natural language processing (NLP) techniques to detect and subsequently redact or substitute identifiers [14], [15], [16], [17]. As demonstrated through systematic reviews [18] and various competitions [19], [20], the most scalable versions of such techniques are rooted in, or rely heavily upon, machine learning methods, in which the publisher of the data annotates instances of personal identifiers in the text, such as patient and doctor name, Social Security Number, and a date of birth, and the machine attempts to learn a classifier (e.g., a grammar) to predict where such identifiers reside in a much larger corpus. Unfortunately, generating a perfectly annotated corpus for training purposes can be extremely costly [21]. This, combined with the natural imperfection of even the best classification learning methods implies that some sensitive information will invariably leak through to the data recipient. This is clearly a problem if, for instance, the information leaked corresponds to direct identifiers (e.g., personal name) or quasi-identifiers (e.g., ZIP codes or dates of birth) which may be exploited in re-identification attacks, such as the re-identification of Thelma Arnold in the search logs disclosed by AOL [22] or the Social Security Numbers in Jeb Bush's emails [23].

**Fig. 1:**
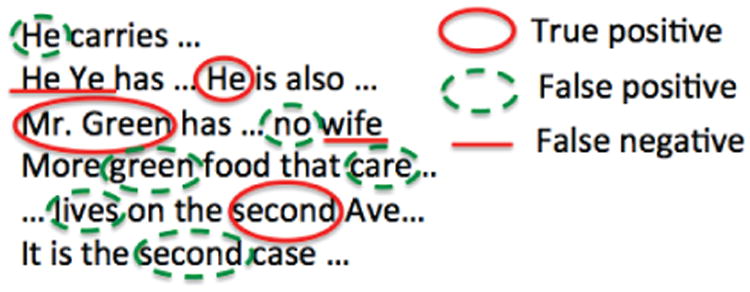
An example of sensitive and non-sensitive instances that need to be distinguished via manual inspection.

Rather than attempt to detect and redact every sensitive piece of information, our goal is to guarantee that even if identifiers remain in the published data, the adversary cannot easily find them. Fundamental to our approach is the acceptance of non-zero privacy risk, which we view as unavoidable. This is consistent with most privacy regulation, such as HIPAA, which allows expert determination that privacy “risk is very small” [2], and the EU Data Protection Directive, which “does not require anonymisation to be completely risk-free” [24]. Our starting point is a threat model within which an attacker uses published data to first train a classifier to predict sensitive entities based on a labeled subset of the data, prioritizes inspection based on the predicted positives, and inspects and verifies the true sensitivity status of *B* of these in a prioritized order. Here, *B* is the budget available to inspect (or read) instances and *true sensitive* entities are those which have been correctly labeled as sensitive (for example, *true sensitive* entities could include identifiers such as a name, Social Security Number, and address). An illustration of such a setting is depicted in Figure 1. In this threat model, we consider an idealized adversary with several elements of omniscience. First, we assume that the adversary can always correctly assess the true sensitivity for any manually inspected instance. Second, we assume that the adversary computes an optimal classifier, that is, a classifier with maximum accuracy within a given hypothesis class, with respect to published data.

We use this threat model to construct a game between a *publisher*, who 1) applies a collection of classifiers to an original data set, 2) prunes all the positives predicted by any classifier, and 3) publishes the remainder, and an *adversary* acting according to our threat model. The data publisher's ultimate goal is to release as much data as possible while at the same time redacting sensitive information to the point where re-identification risk is sufficiently low. In support of the second goal, we show that any locally optimal publishing strategy exhibits the following two properties when the loss associated with exploited personal identifiers is high: *a*) an adversary cannot learn a classifier with a high true positive count, and *b*) an adversary with a large inspection budget cannot do much better than manually inspecting and confirming instances chosen uniformly at random (i.e., the classifier adds little value).

Moreover, we introduce a greedy publishing strategy which is guaranteed to converge to a local optimum and consequently guarantees the above two properties in a linear (in the size of the data) number of iterations. At a high level, the greedy algorithm iteratively executes learning and redaction. It repeatedly learns the classifier to predict sensitive entities on the remaining data, and then removes the predicted positives, until a local optimum is reached. The intuition behind the iterative redaction process is that, in each iteration, the learner essentially checks to determine if an adversary could obtain utility by uncovering residual identifiers; if so, these instances are redacted, while the process is terminated otherwise. Our experiments on two distinct electronic health records data sets demonstrate the power of our approach, showing that 1) the number of residual true positives is always quite small, addressing the goal of reducing privacy risk, 2) confirming that the attacker with a large budget cannot do much better than uniformly randomly choosing entities to manually inspect, 3) demonstrating that most (> 93%) of the original data is published, thereby supporting the goal of maximizing the quantity of released data, and 4) showing that, in practice, the number of required algorithm iterations (< 5) is a small fraction of the size of the data. Additional experiments, involving three datasets that are unrelated to the health domain corroborate these findings, demonstrating generalizability in our approach.

A short version of this paper was presented at the IEEE International Conference on Data Mining [25]. This extended paper offers a number of significant additional contributions, including 1) extended theoretical analysis of locally optimal data publishing policies, 2) finite sample bounds to significantly generalize the theoretical results, and 3) a significantly augmented experimental evaluation.

## 2 Related Work

### Approaches for Anonymizing Structured Data

There has been a substantial amount of research conducted in the field of privacy-preserving data publishing (PPDP) over the past several decades [5], [26]. Much of this work is dedicated to methods that transform well-structured (e.g., relational) data to adhere to a certain criterion or a set of criteria, such as *k*-anonymization [6], *l*-diversity [27], *m*-invariance [28], and *∊*-differential privacy [7], among a multitude of others. These criteria attempt to offer guarantees about the ability of an attacker to either distinguish between different records in the data or make inferences tied to a specific individual. There is now an extensive literature aiming to operationalize such PPDP criteria in practice through the application of techniques such as generalization, suppression (or removal), and randomization (e.g., [29], [30], [31], [32], [33], [34]). All of these techniques, however, rely on *a priori* knowledge of which features in the data are either themselves sensitive or can be linked to sensitive attributes. This is a key distinction from our work: we aim to *automatically discover* which entities in unstructured data are sensitive, as well as formally ensure that whatever sensitive data remains cannot be easily unearthed by an adversary.

### Traditional Methods for Sanitizing Unstructured Data

In the context of privacy preservation for unstructured data, such as text, various approaches have been proposed for the automatic discovery of sensitive entities, such as identifiers. The simplest of these rely on a large collection of rules, dictionaries, and regular expressions (e.g., [35], [36]). [37] proposed an automated data sanitization algorithm aimed at removing sensitive identifiers while inducing the least distortion to the contents of documents. However, this algorithm assumes that sensitive entities, as well as any possible related entities, have already been labeled. Similarly, [38] have developed the *t*-plausibility algorithm to replace the known (labeled) sensitive identifiers within the documents and guarantee that the sanitized document is associated with least *t* documents.

### Machine Learning Methods for Sanitizing Unstructured Data

A key challenge in unstructured data that makes it qualitatively distinct from structured is that even identifying (labeling) which entities are sensitive is non-trivial. For example, while a structured portion of electronic medical records would generally have known sensitive categories, such as a patient's name, physician's notes do not have such labels, even though they may well refer to a patient's name, date of birth, and other potentially identifying information. While rule-based approaches, such as regular expressions, can automatically identify some of the sensitive entities, they have to be manually tuned to specific classes of data, and do not generalize well. A natural idea, which has received considerable traction in prior literature, is to use machine learning algorithms, trained on a small portion of labeled data, to automatically identify sensitive entities. Numerous classification algorithms have been proposed for this purpose, including decision stumps [39], support vector machines (SVM) [40], conditional random fields (CRFs) [14], [17], [41], hybrid strategies that rely on rules and statistical learning models [42], [43] ensemble methods [18]. Unfortunately, such PPDP algorithms fail to formally consider the adversarial model, which is crucial for the decision making of the data publisher. A recent work by Carrell et al. [44] considers enhancing such redaction methods by replacing removed identifiers with fake identifiers which appear real to a human reader.

Our approach builds on this literature, but is quite distinct from it in several ways. First, we propose a novel explicit threat model for this problem, allowing us to make formal guarantees about the vulnerability of the published data to adversarial re-identification attempts. Our model bears some relationship to a recent work by Li et al. [45] who also consider an adversary using machine learning to re-identify residual identifiers. However, our model combines this with a budget-limited attacker who can manually inspect instances; in addition, our publisher model involves the choice of a redaction policy, whereas Li et al. focus on the publisher's decision about the size of the training data, and use a traditional learning-based redaction approach. Second, we introduce a natural approach for sanitizing data that uses machine learning in an iterative framework. Notably, this approach performs significantly better than a standard application of CRFs, which is the leading approach for text sanitization to date [46], but can actually make use of arbitrary machine learning algorithms.

### Game Theory in Security and Privacy

Our work can be seen within the broader context of game theoretic modeling of security and privacy [47], [48], [49], [50], [51], including a number of efforts that use game theory to make machine learning algorithms robust in adversarial environments [52], [53], [54], [55], [56], [57]. In both of these genres of work, a central element is an explicit formal threat (i.e., attacker) model, with the game theoretic analysis generally focused on computing defensive privacy-preserving strategies. None of this work to date, however, addresses the problem of PPDP of unstructured data with sensitive entities not known *a priori*.

## 3 Model

Before delving into the technical details, we offer a brief high-level intuition behind the main idea in this paper.

Suppose that a publisher uses a machine learning algorithm to identify sensitive instances in a corpus, these instances are then redacted, and the residual data is shared with an attacker. The latter, aspiring to uncover residual sensitive instances (e.g., identifiers) can, similarly, train a learning algorithm to do so (using, for example, a subset of published data that is manually labeled). At the high level, consider two possibilities: first, the learning algorithm enables the attacker to uncover a non-trivial amount of sensitive information, and second, the learning algorithm is relatively unhelpful in doing so. In the latter case, the publisher can perhaps breath freely: few sensitive entities can be identified by this attacker, and the risk of published data is low. The former case is, of course, the problem. However, notice that, in principle, the publisher can *try out* this attack in advance of publishing the data, to see whether it can in fact succeed in this fashion. Moreover, if the attacker is projected to be sufficiently successful, the publisher has a great deal to gain by *redacting the sensitive entities an attacker would have found*.

Of course, there is no need to stop at this point: the publisher can keep simulating attacks on the published data, and redacting data labeled as sensitive, until these simulations suggest that the risk is sufficiently low. This, indeed, is the main idea. However, many details are clearly missing: for example, what does an attacker do after training the learning algorithm, when, precisely, should the publisher stop, and what can we say about the privacy risk if data is published in this manner, under this threat model? Next, we formalize this idea, and offer precise answers to these and other relevant questions.

Table 1 summarizes the notation used throughout this paper. Imagine that a publisher's dataset consists of a set of *n* entities (or words), *X* = {*x*_1_, …, *x_n_*}, of which he will publish a subset *P* ⊆ *X*. The publisher may have an additional data set for training a classifier to predict whether an entity *x* is sensitive. We let *α* denote the fraction of the original *n* entities that are sensitive. A learning algorithm is designed to select a hypothesis that best supports the data. Here we consider the hypothesis to be a function *f* mapping from the data space *𝒟* to the response space *ε*; i.e., *f* : *𝒟* → *ε*. Of course there are many such hypotheses. We assume *f* belongs to a family of hypotheses *ℋ*. Specifically the response space *ε* = {0, 1} within our problem indicates whether the entity *x* is sensitive (*S*, *f*(*x*) = 1) or non-sensitive (*N*, *f*(*x*) = 0), and *ℋ* represents a set of binary classifiers.

A crucial assumption in our approach is that the hypothesis class *ℋ* is known to both the publisher and attackers. This is a natural assumption, considering that state-of-the-art machine learning algorithms are well-known and typically have multiple high-quality open source implementations. Moreover, even as new approaches are developed for identifying sensitive entities in unstructured (e.g., text) data, these approaches can be subsequently incorporated into our framework. Note that our assumption of common knowledge of *ℋ* does *not* imply that the publisher knows the actual function *f* used by the attacker (see threat model below). The importance of this point is highlighted when we analyze finite sample bounds in Section 4.

We use *h* to denote a classifier chosen from the hypothesis class *ℋ*. For a classifier *h* and a data set *Y*, we introduce the following notation:

- *FP*(*h*, *Y*) = | ⋃_*x∈Y*_ {*x* ∈ *N*|*h*(*x*) = 1}|: the number of false positive instances of *h* on *Y*;
- *TP*(*h*, *Y*) = | ⋃_*x∈Y*_ {*x* ∈ *P*|*h*(*x*) = 1}|: the number of true positive instances of *h* on *Y*;
- *FN*(*h*, *Y*) = | ⋃_*x∈Y*_ {*x* ∈ *P*|*h*(*x*) = 0}|: the number of false negative instances of *h* on *Y*; and
  *TN*(*h*, *Y*) = | ⋃_*x∈Y*_ {*x* ∈ *N*|*h*(*x*) = 0}|: the number of true negative instances of *h* on *Y*.

Clearly, if |*Y*| = *m*, then *FP*(*h*, *Y*)+*TP*(*h*, *Y*)+*FN*(*h*, *Y*)+*TN*(*h*, *Y*) = *m* ∀*h* ∈ *ℋ*. Finally, we define *FP*(*h*, ∅) = *FN*(*h*, ∅) = *TP*(*h*, ∅) = *TN*(*h*, *∅*) ≡ 0.

### Threat Model

Suppose that an adversary obtains the published data *P* ⊆ *X*. We assume that an adversary has a fixed inspection budget, *B*, which can be thought of as manual inspection of actual instances to verify whether or not they are sensitive (and, consequently, have value to the adversary). If a sensitive instance is found, we assume the adversary gains *L*, which is identical to the publisher's loss. Thus, when the attacker selects a set *I* ⊆ *P* of instances for inspection, such that |*I*| ≤ *B*, his utility is


(1) $$U_{A}(I)=L|\{\text{sensitive instances}\in I\}|=L\sum_{x\in I}S(x),$$

where *S*(*x*) = 1 iff *x* is sensitive. A central aspect of the threat model is the specific way that the attacker chooses the set *I* of instances to inspect. A simple baseline is to choose *I* uniformly at random from *P*. We use *U_A_* to denote the utility that the attacker obtains when using this simple baseline. Presumably, however, the attacker can do better by using a more sophisticated strategy. In particular, we suppose that a *sophisticated* attacker proceeds as follows:

1. Choose a classifier
(2) $$h_{A}(P)\in \arg\min_{h\in ℋ}\frac{FP(h,P)+FN(h,P)}{\left|P\right|}.$$
  In other words, the attacker chooses an optimal classifier from *ℋ* in terms of accuracy. From the publisher's perspective, this is a very pessimistic limit of an attacker who uses a subset of *P* for training a standard classification algorithm, such as an SVM.
2. Prioritize instances in *P* by ranking all *x* ∈ *P* with h*(*x*) = 1 first, followed by those with h*(*x*) = 0. Within each class, the order is arbitrary.
3. Choose *I* in this ranked order until it contains *B* instances. In other words, first the attacker will choose the predicted positives, followed by predicted negatives (if there is any budget remaining).

We simply refer to *h_A_* where *P* is clear from context. We let $U_{A}^{*}$ denote the attacker's utility when using this more sophisticated learning-based strategy. A technical caveat is that, depending on the quality of the classifier, $U_{A}^{*}$ is not necessarily higher than *U_A_*. Below, we provide a sufficient condition for $U_{A}^{*}\geq U_{A}$.

As an illustration, let us return to Figure 1, which presents an example of the behavior of an attacker given a published dataset containing sensitive and non-sensitive instances. Assume the circled words are classified as positives by *h_A_*. The attacker would inspect these words and their surrounding context first. However, in this setting, some of the words inspected are not sensitive instances (i.e., false positives; shown in dashed ovals). For example, the first dashed “He” is a pronoun, while the solid circled “He” is actually the name of a person. Therefore, if the attacker has sufficient budget to inspect all of the circled instances, he would gain 3 units of utility (i.e., true positives, shown in solid ovals), and waste 3 units of budget (again, in dashed ovals).

### Data Publisher Model

To develop some intuition for our publisher model, let us first consider the typical approach for sanitizing data (we assume for now that the defender is able to learn an optimal classifier; we relax this assumption below):

1. Learn a classifier
(3) $$\bar{h}\in \arg\min_{h\in ℋ}\frac{FP(h,X)+FN(h,X)}{\left|X\right|}.$$
  Let *X*_1_ = {*x* ∈ *X*|*h̄*(*x*) = 1} (i.e., *X*_1_ is the set of predicted positives).
2. Publish the data set *P* = *X* \ *X*_1_.

Essentially all of the approaches in the literature assume this, or a similar, form. To apply our threat model above, we consider two possibilities: a) the attacker's classifier *h_A_* can successfully identify residual sensitive instances, or b) the attacker's classifier cannot detect residual positives. If we are in situation (b), the publisher can view the sanitization as a success. Situation (a), on the other hand, is clearly problematic, but it also suggests a natural solution: the publisher can apply *h_A_* to residual data, remove the sensitive instances, and only then publish the data. Indeed, this is where the symmetry between the publisher and attacker, taking advantage of the common knowledge of *ℋ*, is pivotal. Specifically, *the publisher can simulate anything that the attacker would do*.

Moreover, there is no reason to stop at this point. In fact, the publisher should continue as long as the simulated classifier that would be used by the attacker is sufficiently good. This observation also offers the key intuition for our results. Whenever the publisher chooses to stop, the attacker's ability to identify sensitive instances must inherently be relatively weak. Of course, this will depend on the relative loss to the publisher from correctly identified sensitive entities and the value of publishing data.

**Fig. 2:**
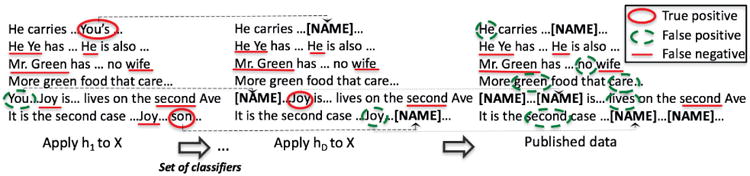
The process for applying a set of classifiers *H* to data *X*.

Using the developed intuition, we model the publisher as selecting a finite set of classifiers *H* ⊆ ℋ, where *H* = {*h*_1_, *h*_2_, …, *h_D_*}. Figure 2 shows the process of generating and publishing the data in Figure 1. After applying each classifier *h_i_*, the positive instances are replaced with the fake tokens, such as “**[NAME]**” replacing an individual's name.

Let *X*_1_(*H*) = ⋃_*h*∈*H*_{*x* ∈ *X*|*h*(*x*) = 1}, that is, the set of all positives predicted by the classifiers in *H*, and let *P*(*H*) = *X* \ *X*_1_(*H*); we use *P* with no argument where *H* is clear from context. The publisher's approach is:

1. Choose a collection of classifiers *H* (we address this choice below).
2. Publish the data set *P*(*H*) = *X* \ *X*_1_(*H*).

Let *FN*(*H*) be the number of false negatives of *H* in *X*, which we define as all residual sensitive instances in *P*, and let *FP*(*H*) be the number of false positives in *X*, that is, all predictive positives by any *h* ∈ *H* which are, in fact, not sensitive. It directly follows that for any *H*, *FN*(*H*) ≤ *αn* (i.e., the number of false negatives is, at most, the total number of sensitive entities in the original data) and *TN*(*H*) ≤ (1 – *α*)*n* (i.e., the number of true negatives is, at most, the total number of non-sensitive entities). If we allow the attacker to have an infinite budget, then every false negative will be exploited, resulting in the total loss of *L* · *FN*(*H*). In addition, each false positive costs the publisher a fixed amount *C*, which we can interpret as the value of publishing the data. Thus, we define the (worst-case) total loss to the publisher from using a set of classifiers *H* as


(4) T(H)=L⋅FN(H)+C⋅FP(H),

where *FN*(*H*) = | ⋂_*h*∈*H*_ {*x* ∈ *S*|*h*(*x*) = 0}|, *FP*(*H*) = | ⋃_*h*∈*H*_ {*x* ∈ *N*|*h*(*x*) = 1}|, and *S*, *N* represent the sensitive and non-sensitive instances, respectively. *TN*(*H*) and *TP*(*H*) are defined similarly.

### Contextual Information and Inference Attacks

A significant amount of work in privacy and data sanitization deals with linkage attacks [58], [59], [60]. Of particular relevance to our purpose are correlations among words in documents which enable an attacker to recover some sensitive information that has been removed [16]. Our methods can be extended directly to consider contextual information in two ways. First, we can use previous methods to discover entities in training data correlated with identifiers, and label these as identifiers as well. We can then apply our methods separately for different categories of identifiers as well as derived (correlated) words and phrases to remove both identifying information and any contextual data. Alternatively, we can first apply our methods to learn a collection of classifiers predicting identifiers in test data, and use association-based methods, such as [16], to remove additional contextual information from the test data. Henceforth, we focus on the core problem of predicting identifiers.

## 4 A Greedy Algorithm for Automated Data Sanitization

Given a formal model, we can now present our iterative algorithm for automated data sanitization, which we term *GreedySanitize*. Our algorithm (shown as Algorithm 1) is simple to implement and involves iterating over the following steps: 1) compute a classifier on training data, 2) remove all predicted positives from the training data, and 3) add this classifier to the collection. The algorithm continues until a specified stopping condition is satisfied, at which point we publish only the predicted negatives, as above. While the primary focus of the discussion so far, as well as the stopping criterion, have been to reduce privacy risk, the nature of *GreedySanitize* is to also preserve as much utility as feasible: this is the consequence of stopping as soon as the re-identification risk is minimal.


**Algorithm 1** GreedySanitize(*X*), *X* : training data.
 *H* ← {}, *k* ← 0, *h*_0_ ← ∅, *D*_0_ ← *X*, **repeat**  *H* ← *H* ⋃ *h_k_*  *k* = *k* + 1  *h_k_* ← LearnClassifier(*D_k_*_–1_)  *D_k_* ← RemovePredictedPositives(*D_k_*_–1_, *h_k_*) **until**
*T*(*H* ⋃ *h_k_*) – *T*(*H*) ≥ 0 **return**
*H*


It is important to emphasize that *GreedySanitize* is *qualitatively different* from typical ensemble learning schemes in several ways. First, a classifier is retrained in each iteration on data that includes only predicted negatives from all prior iterations. To the best of our knowledge this is unlike the mechanics of any ensemble learning algorithm.^1^ Second, our algorithm removes the union of all predicted positives, whereas ensemble learning typically applies a weighted voting scheme to predict positives; our algorithm, therefore, is fundamentally more conservative when it comes to sensitive entities in the data. Third, the stopping condition is uniquely tailored to the algorithm, which is critical in enabling provable guarantees about privacy-related performance.

Given the iterative nature of the algorithm, it is not obvious that it will terminate. The following theorem asserts that *GreedySanitize* will always terminate in a linear number of iterations.

### Theorem 1

Algorithm 1 terminates after at most |*X*| iterations, where *X* is the set of entities in the training data.

*Proof.* Let *TP*(*D_i_*), *FP*(*D_i_*), *TN*(*D_i_*), and *FN*(*D_i_*) specifically refer to these quantities computed on *training data D_i_* remaining in iteration *i* of the algorithm. Suppose that there exists an iteration *i* such that *TP*(*D_i_*_–1_) = 0. It is clear that Algorithm 1 will stop after this iteration. Now, suppose instead that *TP*(*D_i_*_–1_) ≥ 1 in every iteration. In this case, in at most |*X*| iterations no data will remain, and *TP*(∅) = 0 by definition. Consequently, either *TP*(*D_i_*_–1_) = 0 for *i* < |*X*| and the algorithm will terminate, or the algorithm will stop when *i* = |*X*|.

Next, we provide additional theoretical analysis of the proposed *GreedySanitize* algorithm focusing on two questions. First, what kinds of privacy guarantees does this algorithm offer? Second, how can we generalize the privacy guarantees to account for finite sample approximations inherent in the algorithm? To address the first question, we abstract away the details of our algorithm behind the veil of its stopping condition, which turns out to be the primary driver of our results. This also allows us to state the privacy guarantees in much more general terms.

### Analysis of Locally Optimal Publishing Policies

In this section we analyze the adversary's ability to infer sensitive information from published data if the defender's choice of classifiers *H* to apply to original data satisfies the following *local optimality* condition.

#### Definition 4.1

A set of classifiers *H* ⊆ ℋ is a local optimum if *T*(*H* ⋃ *h_A_*) – *T*(*H*) ≥ 0.

In plain terms, a subset is a local optimum if the adversary's optimal classifier *h_A_* (that is, the attacker's best classifier choice to apply to the published data), when added to this subset, does not improve the publisher's utility. Under a minor regularity condition that ℋ contains an identity (which can always be added), there is always a trivial local optimum of not releasing any data. Notice that the local optimality condition is exactly the stopping condition of *GreedySanitize*. This means that, when the algorithm terminates, its output set of hypotheses *H* is guaranteed to be a local optimum.

We now present a lemma that enables us to characterize *all of the local optima*.

#### Lemma 1

For an arbitrary set of classifiers *H* ⊆ ℋ,

1. *FN*(*H*) = *FN*(*H* ⋃ *h*) + *TP*(*h*, *P*(*H*)), *and*
2. *FP*(*H* ⋃ *h*) = *FP*(*H*) + *FP*(*h*, *P*(*H*)).

*Proof.* For result 1, define the set


$$\tilde{FN}(H)=\cap_{\tilde{h}\in H}\{x\in S|\tilde{h}(x)=0\}.$$

Thus,


$$\tilde{FN}(H\cup h)=\cap_{\tilde{h}\in H}\{x\in S|\tilde{h}(x)=0\}\cap \{x\in S|h(x)=0\}.$$

We can represent $\tilde{FN}(H)$ as


$$\begin{array}{c} \tilde{FN}(H)=(\tilde{FN}(H)\cap \{x\in S|h(x)=0\})\cup (\tilde{FN}(H)\cap \{x\in S\backslash h(x)=1\})\\ =\tilde{FN}(H\cup h)\cup (\tilde{FN}(H)\cap \{x\in S|h(x)=1\}) \end{array}.$$

Moreover, note that $x\in \tilde{FN}(H)$ implies that *x* ∈ *P*(*H*), so that


$$\begin{array}{c} \tilde{FN}(H)=\tilde{FN}(H\cup h)\cup (\tilde{FN}(H)\cap \{x\in P(H)\cap S|h(x)=1\})\\ =\tilde{FN}(H\cup h)\cup \tilde{TP}(h,P(H)), \end{array}$$

where $\tilde{TP}(h,P(H))$ is the set of all true positives of *h* on *P*(*H*). Moreover, by definition these two sets are non-overlapping, and thus


FN(H)=FN(H∪h)∪TP(h,P(H)).

For result 2, define the set


$$\tilde{FP}(H)=\cup_{\tilde{h}\in H}\{x\in N|\tilde{h}(x)=1\}.$$

Therefore,


$$\begin{array}{c} \tilde{FP}(H\cup h)=\cup_{\tilde{h}\in H}\{x\in N|\tilde{h}(x)=1\}\cup \{x\in N|h(x)=1\}\\ =\tilde{FP}(H)\cup \{x\in N|h(x)=1\}. \end{array}$$

By definition, *x* ∈ *N* and *x* ∉ *P*(*H*) means that $x\in \tilde{FP}(H)$. Thus,


$$\begin{array}{c} \tilde{FP}(H\cup h)=\tilde{FP}(H)\cup \{x\in N\cap P(H)|h(x)=1\}\\ =\tilde{FP}(H)\cup \tilde{FP}(h,P(H)). \end{array}$$

Moreover, $x\in \tilde{FP}(H)$ means that *x* ∉ *P*(*X*), so that these two subsets do not overlap, and we thus obtain


FP(H∪h)=FP(H)+FP(h,P(H)).

We can now state the primary result, which characterizes all locally optimal solutions *H*.

#### Theorem 2

*H* ⊆ ℋ is a local optimum if and only if either *TP*(*h_A_*, *P*) = 0 or $\frac{FP(h_{A},P)}{TP(h_{A},P)}\geq \frac{L}{C}$.

*Proof.* By definition, *H* is a local optimum if, and only if,


$$L(FN(H\cup h_{A})-FN(H))+C(FP(H\cup h_{A})-FP(H))\geq 0.$$

By Lemma 1, *FN*(*H* ⋃ *h_A_*) – *FN*(*H*) = −*TP*(*h_A_*, *P*) and *FP*(*H* ⋃ *h_A_*) – *FP*(*H*) = *FP*(*h_A_*, *P*), so that a local optimum is characterized by


$$C\cdot FP(h_{A},P)\geq L\cdot TP(h_{A},P).$$

If *TP*(*h_A_*, *P*) = 0, this inequality clearly holds. Suppose that *TP*(*h_A_*, *P*) ≥ 1. In this case, by rearranging the expression, it can be seen that *H* is a local optimum if, and only if, $\frac{FP(h_{A},P)}{TP(h_{A},P)}\geq \frac{L}{C}$.

Below, we simplify notation by defining *FP_a_* ≡ *FP*(*h_A_*, *P*), and defining *FN_A_ TP_A_*, and *TN_A_* similarly, with *H* becoming an implicit argument throughout. Now, observe that if *L/C* > (1 – *α*)*n*, the only locally optimal solutions have *TP_A_* = 0, because otherwise $\frac{FP_{A}}{TP_{A}}\leq (1-\alpha )n<L/C$.

As a direct consequence of Theorem 2, we can bound *TP_A_* in all locally optimal solutions.

#### Theorem 3

*For any locally optimal H* ⊆ ℋ, $TP_{A}\leq \frac{C}{L}(1-\alpha )n$.

*proof.* If *TP_A_* = 0, the result is trivially true. Suppose *TP_A_* ≥ 1. Then, since $\frac{FP_{A}}{TP_{A}}\geq \frac{L}{C}$, we have $TP_{A}=TP_{A}\leq \frac{C}{L}FP_{A}\leq \frac{C}{L}TN(H)\leq \frac{C}{L}(1-\alpha )n$.

The upshot of Theorem 3 is that when *C* is small relative to *L*, any locally optimal *H* will guarantee that the attacker cannot learn a classifier that correctly identifies more than a few sensitive instances. This result further implies that an attacker with a small budget *B* ≤ *TP_A_* + *FP_A_* (i.e., budget is exceeded by the total number of predicted positives) can obtain very little utility from using the classifier in this case.

But what about attackers with a large budget, such as when *B* ≥ *TP_A_* + *FP_A_*? Clearly, when the budget is sufficiently large, the attacker will identify all the residual sensitive information in the data. However, we now show that, even in this case, an attacker can do little better than the trivial baseline of choosing *B* instances to inspect in a uniformly at random manner. An important technical consideration is that when *TP_A_* = 0, an adversary can actually improve performance by prioritizing the negative predictions over the predicted positives (which yield no utility). In this case, an adversary will likely throw away the classifier altogether. We therefore restrict our attention to the case when the attacker actually benefits from prioritizing positives over negatives. The following lemma provides a sufficient condition for this observation.

#### Lemma 2

Let *B* ≥ *TP_A_* + *FP_A_*. When *TP_A_TN_A_* ≥ *FP_A_FN_A_*, prioritizing positive over negative instances guarantees that $U_{A}^{*}\geq U_{A}$ for the attacker.

*proof.* If the attacker prioritizes negatives before positives, the attacker's utility is


$$U_{A*}=L\cdot (FN_{A}+\frac{TP_{A}}{TP_{A}+FP_{A}}(B-FN_{A}-TN_{A})),$$

whereas the utility from the uniform random baseline is


$$U_{A}=L\cdot \frac{TP_{A}+FN_{A}}{TP_{A}+FP_{A}+TN_{A}+FN_{A}}B.$$

Thus, when *TP_A_TN_A_* ≥ *FP_A_FN_A_*,


$$\begin{array}{c} \frac{U_{A*}}{U_{A}}=\frac{FP_{A}FN_{A}+TP_{A}B-TP_{A}TN_{A}}{B}(\frac{TP_{A}+FP_{A}+FN_{A}+TN_{A}}{\left(TP_{A}+FN_{A})(TP_{A}+FP_{A}\right)})\\ =(\frac{FP_{A}FN_{A}-TP_{A}TN_{A}}{B}+TP_{A})(\frac{\left(TP_{A}+FP_{A}+FN_{A}+TN_{A}\right)}{\left(TP_{A}+FN_{A})(TP_{A}+FP_{A}\right)})\\ \leq (\frac{FP_{A}FN_{A}-TP_{A}TN_{A}}{TP_{A}+aFP_{A}}+TP_{A})(\frac{\left(TP_{A}+FP_{A}+FN_{A}+TN_{A}\right)}{\left(TP_{A}+FN_{A})(TP_{A}+FP_{A}\right)})\\ =1+\frac{\left(FP_{A}FN_{A}-TP_{A}TN_{A})(FN_{A}+TN_{A}\right)}{{\left(TP_{A}+FP_{A}\right)}^{2}(TP_{A}+FN_{A})}\leq 1. \end{array}$$

Since *U_A_* cannot be larger than both the utility from prioritizing positive prioritizing negative instances (being the average of these), the result follows.

Under the condition in Lemma 2, we can now prove a bound on the the amount that the attacker can gain over the trivial baseline by using a classifier to prioritize instances, or the ratio $U_{A}^{*}/U_{A}$.

#### Theorem 4

Suppose that H is a local optimum, the attacker's budget is *B* ≥ *TP_A_* + *FP_A_*, and *TP_A_TN_A_* ≥ *FP_A_FN_A_*. Then


$$\frac{U_{A}^{*}}{U_{A}}\leq \frac{(1-\alpha )n+1}{1+\frac{L}{C}}.$$

In order to prove this theorem, we need another building block, provided by the following Lemma.

#### Lemma 3

Suppose that *B* ≥ *TP_A_* + *FP_A_*, *TP_A_TN_A_* ≥ *FP_A_FN_A_*, and the attacker prioritizes positive instances. Then


$$\frac{U_{A}^{*}}{U_{A}}\leq 1+\frac{TP_{A}TN_{A}-FP_{A}FN_{A}}{\left(TP_{A}+FP_{A})(TP_{A}+FN_{A}\right)}.$$

*Proof.* Suppose that the attacker prioritizes positives before negatives. Then the attacker's utility is


$$U_{A*}=L(TP_{A}+\frac{FN_{A}}{FN_{A}+TN_{A}}(B-TP_{A}-FP_{A})).$$

Thus,


$$\begin{array}{c} \frac{U_{A*}}{U_{A}}=\frac{TP_{A}TN_{A}+FN_{A}B-FP_{A}FN_{A}}{B}(\frac{TP_{A}+FP_{A}+FN_{A}+TN_{A}}{\left(TP_{A}+FN_{A})(TN_{A}+FN_{A}\right)})\\ =(\frac{TP_{A}TN_{A}-FP_{A}FN_{A}}{B}+FN_{A})(\frac{TP_{A}+FP_{A}+FN_{A}+TN_{A}}{\left(TP_{A}+FN_{A})(TN_{A}+FN_{A}\right)})\\ \leq (\frac{TP_{A}TN_{A}-FP_{A}FN_{A}}{TP_{A}+FP_{A}}+FN_{A})(\frac{TP_{A}+FP_{A}+FN_{A}+TN_{A}}{\left(TP_{A}+FN_{A})(TN_{A}+FN_{A}\right)})\\ =1+\frac{TP_{A}TN_{A}-FP_{A}FN_{A}}{\left(TP_{A}+FP_{A})(TP_{A}+FN_{A}\right)}. \end{array}$$

*Proof.* **of Theorem 4** Since *TP_A_TN_A_* ≥ *FP_A_FN_A_*, the attacker will prioritize positive instances by Lemma 2. Therefore, by Lemma 3,


$$\begin{array}{c} \frac{U_{A*}}{U_{A}}\leq 1+\frac{TP_{A}TN_{A}-FP_{A}FN_{A}}{\left(TP_{A}+FP_{A})(TP_{A}+FN_{A}\right)}\\ =1+\frac{TN_{A}-\frac{FP_{A}}{TP_{A}}\cdot FN_{A}}{\left(1+\frac{FP_{A}}{TP_{A}})(TP_{A}+FN_{A}\right)}\\ \leq 1+\frac{TN_{A}-\frac{L}{C}\cdot FN_{A}}{\left(1+\frac{L}{C})(TP_{A}+FN_{A}\right)}\leq 1+\frac{(1-\alpha )n-\frac{L}{C}}{1+\frac{L}{C}}\\ =\frac{(1-\alpha )n+1}{1+\frac{L}{C}}. \end{array}$$

The upshot of Theorem 4 is that even an attacker with a large budget cannot do much better than uniformly selecting instances to inspect.

#### Example 1

As an example, again consider Figure 1, which illustrates the result after the application of the set of classifiers H. It can be seen that there are 26 instances in total, with a breakdown of 3 true positives, 6 false positives, 15 true negatives, and 2 false negatives. Now, if the attacker has a budget of *B* = 20, $\frac{U_{A*}}{U_{A}}=\frac{3+(20-3-6)\frac{2}{2+15}}{20\frac{3+2}{26}}\approx 1.11$.

### Finite Sample Bounds

Armed with the idealized generic analysis of locally optimal classifier subsets *H*, we can generalize these results to account for finite sampling error. While the results in the previous section are applicable for arbitrary locally optimal subsets, our finite sample analysis is specific to *GreedySanitize*.

Consider the point at which the publisher halts the greedy data sanitization Algorithm 1 and publishes the data (after applying the resulting set of classifiers *H*). If only a few training data points remain, the publisher's decision would entail significant risk because the error in estimating the relevant decision parameters will be quite high. As such, in this case, no data should be published. We therefore consider the case when there is a non-trivial amount of training data remaining after Algorithm 1 terminates. As our experiments below demonstrate, this is a reasonable assumption to invoke in practice. In the following discussion, we denote the size of this residual training data *m*.^2^

Our point of departure is the standard learning-theoretic framework. To simplify the presentation, we assume that the published data set is sufficiently large, so that the relevant quantities (e.g., the number of true positives) are close to their expected values on randomly chosen data sets of the same size. Now, let our hypothesis class ℋ contain a set of functions from a set *X* to {0,1}, and assume ℋ has finite Vapnik-Chervonenkis dimension *υ* ≥ 1. Suppose that *P* is the data set remaining after Algorithm 1 terminates and the resulting classifiers *H* are applied to the original data *X*. Let the classifier used in the last iteration by Algorithm 1 be $\hat{h_{A}}$, which is only optimal on training data. In other words, $\hat{h_{A}}$ is the publisher's approximation of the classifier *h_A_* that would subsequently be applied by the attacker to *P*. Let $\hat{FN_{A}}$, $\hat{FP_{A}}$, $\hat{TP_{A}}$, $\hat{TN_{A}}$ be the corresponding approximate counts of false negatives, false positives, etc., applying $\hat{h_{A}}$ to the training data, whereas *FN_A_*, *FP_A_*, *TP_A_*, and *TN_A_* still denote the corresponding counts for the actual optimal classifier *h_A_* that the attacker would use. The attacker's corresponding utility, estimated using the training data, is denoted by $\hat{U_{A}^{*}}$, while the actual attacker utility is $U_{A}^{*}$. The utility for the attacker gained from the baseline policy is still *U_A_*.

We start by noting the well-known error bound connecting empirical and actual errors in classification:


(5) $$\frac{\hat{FP_{A}}+\hat{FN_{A}}}{m}\leq \frac{FP_{A}+FN_{A}}{m}+\lambda (\delta ,m)$$

with probability at least 1 – *δ*, where


$$\lambda (\delta ,m)={\left(\frac{41}{m}(\upsilon \log(\frac{2em}{\upsilon })+\log(\frac{4}{\delta }))\right)}^{\frac{1}{2}}.$$

For our purposes, however, this result is not sufficient. For example, there may be two classifiers, *h* and *h′* in ℋ with a similar error, but with very different numbers of false positives and false negatives. Thus, in order to bound the utility of the attacker, we need to call upon several additional assumptions. Specifically, we make the following assumptions: $\hat{FP_{A}}\leq p\hat{FN_{A}}$, $\hat{TP_{A}}\geq q\hat{N_{A}}$, *FP_A_* ≥ *sFN_A_*, and *TP_A_* ≤ *rN_A_*. Since the parameters *p*, *q*, *s*, *r* can be arbitrary, these relationships are quite general. However, the results below are most meaningful if these bounds are tight.

#### Lemma 4

Suppose that $\hat{TP_{A}}\geq 1$ when Algorithm 1 terminates. Then,


$$\frac{FP_{A}}{TP_{A}}\geq (\frac{1}{1+\frac{1}{s}})((1+\frac{1}{p})\cdot q\cdot \frac{L}{C}-\lambda (m,\delta ))\frac{1}{r}$$

with probability at least 1 – *δ*.

Clearly, the bound in Lemma 4 is only meaningful when $\lambda (m,\delta )\leq (1+\frac{1}{p})q\frac{L}{C}$, that is, for a sufficiently large sample *m*. Therefore, the results below assume this to be the case.

Building on the result in Lemma 4, we can now extend the bounds on the attacker's success developed in Section 4 to account for finite sample error.

#### Theorem 5

When Algorithm 1 terminates,


$$TP_{A}\leq r(1+\frac{1}{s})\frac{(1-\alpha )n}{\left(1+\frac{1}{p})\cdot q\cdot \frac{L}{C}-\lambda (m,\delta \right)}$$

with probability at least 1 – *δ*.

#### Theorem 6

Suppose that *TP_A_TN_A_* ≥ *FP_A_FN_A_*, and *B* ≥ *TP_A_* + *FP_A_*. Then,


$$\frac{U_{A}^{*}}{U_{A}}\leq \frac{\left((1-\alpha )n+1)r(1+\frac{1}{s}\right)}{r(1+\frac{1}{s})+(1+\frac{1}{p})q\frac{L}{C}-\lambda (\delta ,m)}$$

with probability at least 1 – *δ*.

Proofs of these results are provided in the appendix.

## 5 Experiments

In this section, we assess the performance of *GreedySanitize* (GS) on 5 data sets. Two of these are electronic health record data sets where the goal is to protect personal identifiers; here we only consider the individuals' names: 1) publicly accessible medical records from the I2B2 corpus [19] and 2) a private electronic medical records (EMR) dataset from the Vanderbilt University Medical Center (VUMC). In addition, we evaluate the performance of our model on three more general data sets to assess its generalizability: 1) Enron email Corpus, 2) newsgroup Corpus [62] and 3) Reuters Corpus [63]. In all of these, we also treat individuals' names as sensitive entities. The statistics in Table 2 provide some intuition into the size and complexity of these resources.

Within the i2b2 corpus, we have the synthetic names in place of actual patient identifiers labeled as sensitive instances; while we have the real patient identifiers labeled as sensitive in VUMC. User names in Enron, Newsgroup and Reuters are also labeled as sensitive instances here. We used four state-of-the-art learning algorithms for sensitive entity recognition. The first is conditional random fields (CRF), which consistently ranks as the best method for identifying personal health information in electronic medical records [14], [19], [20]. The second is support vector machine (SVM) [64], which makes use of the features of the word itself, part-of-speech (POS), morphologic information, and the history class of preceding words assigned by the classifier. The third is AdaBoost [65], [66] which reweights the contribution of different data instances. The fourth is a recently proposed ensemble method [18], which applies CRF to classify first and then uses SVM to reduce the false positives.

Each of these approaches play a dual-role in our experiments. First, they serve as a comparison baseline. Second, they function as the core learning algorithms in our own Algorithm 1 (GS). In all the experiments, the attacker first runs all four of these algorithms on the training holdout from published data, and then chooses the best performing classifier. Our evaluation is based on four-fold cross-validation, with GS running on the training data. Note that GS uses the incidence of true and false negatives on the training data to determine when to terminate.

### Privacy Risk

**Fig. 3:**
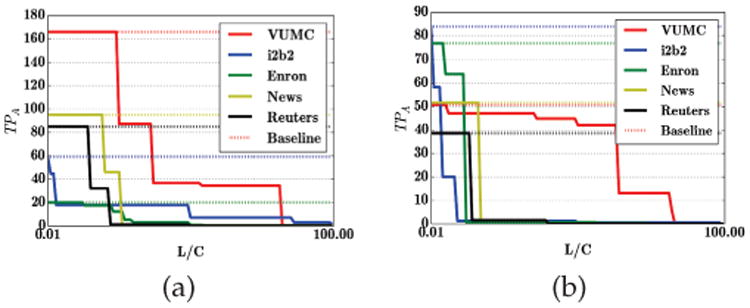
The number of residual *true positive* instances *TP_A_*, which is equivalently the identified instances for an attacker with a small budget after running GS for the i2b2, VUMC, Enron, Newsgroup, and Reuters datasets. We evaluate (a) GS using CRF; (b) GS using the best classifier from {CRF, SVM, AdaBoost, Ensemble}. The dashed lines correspond to the baseline application of the best classifier from this collection.

When the budget of the attacker is small, our theoretical results provide an upper bound on the expected number of identified instances. While this bound suggests that risk becomes arbitrarily small when the associated loss is large, it is not tight. In Figure 3 we demonstrate that the number of identified instances (which is equivalent to the number of true positives for the attacker's classifier) typically becomes negligible even when *L* is quite small relative to *C*. An interesting exception is the VUMC dataset, where the number of identified instances remains relatively large until the loss from re-identification is quite high.

**Fig. 4:**
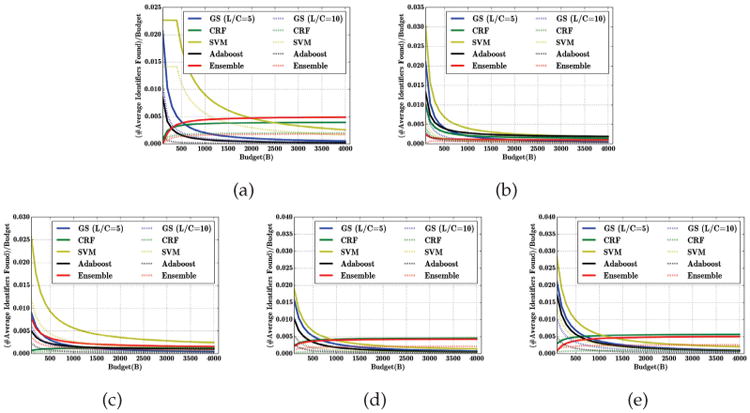
The ratio of the average number of sensitive identifiers found by the attacker and the adversarial budget, while the publisher applies different classifiers with cost sensitive learning with *L/C* ∈ {5, 10}. (a) i2b2, (b) VUMC, (c) Enron, (d) Newsgroup, (e) Reuters datasets.

To investigate privacy risk more generally, we now consider the expected number of identified instances as a function of adversary's budget (and normalized by the budget). To make a meaningful comparison to the state of the art classification schemes, we apply them in a cost sensitive manner, so that *L* becomes the cost of false negatives and *C* the cost of false positives, just as in our model. Figure 4 compares the GS algorithm to the cost sensitive state-of-the-art CRF, SVM, Adaboost, and Ensemble algorithms using the same values of *L* and *C* in GS and cost sensitive versions of the classifiers, respectively. We can see that, for the same values of *L/C*, the GS algorithm is consistently competitive with, or better than, the best state-of-the-art cost sensitive alternatives in terms of privacy risk, except when adversary's budget is extremely small. However, with a small budget, the privacy risk is negligible for sufficiently high *L/C* (Figure 3).

### Data Utility

Next, we investigated the extent to which data utility can be retained in the face of a high privacy requirement. This served as motivation for GS (in comparison to simply suppressing all data), but we did not explicitly consider it in the theoretical analysis. Intuitively, GS should strike a reasonable balance: it stops immediately after a local optimum is reached. In our model, of course, there may be multiple local optima thereafter, but these would result in less data being published. Here, we evaluate the data utility of the published data using the *publish ratio*, which is defined as the proportion of the original number of entities in the published data.

**Fig. 5:**
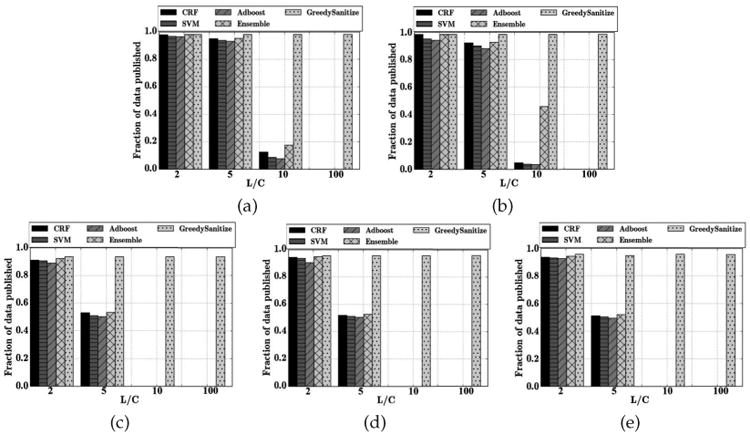
Fraction of data published for different classifiers with cost sensitive learning. (a) i2b2, (b) VUMC, (c) Enron, (d) Newsgroup, and (e) Reuters datasets.

Figure 5 compares GS to cost-sensitive variants of the baseline algorithms (CRF, SVM, Adaboost, and Ensemble). GS preserves most of the data utility even when *L/C* is high. Specifically, in both of the EMR datasets over 98% of the data is published, *even when L/C is quite high*. The performance for the other three data sets is lower, but still, over 93% of the data is ultimately published, even with large *L/C* ratios. In contrast, when the loss due to re-identification is moderate or high, cost-sensitive algorithms essentially suppress most of the data, resulting in very low utility. GS therefore offers a far better balance between risk and utility than the state-of-the-art alternatives.

### Impact of the Size of the Hypothesis Space

When applying GS, it is important to consider that perhaps the attacker may use a new algorithm that the publisher did not considered. We now explore this issue by considering the quality of decisions when the publisher uses only a single classifier or the best of all four, at the core of GS.

**Fig. 6:**
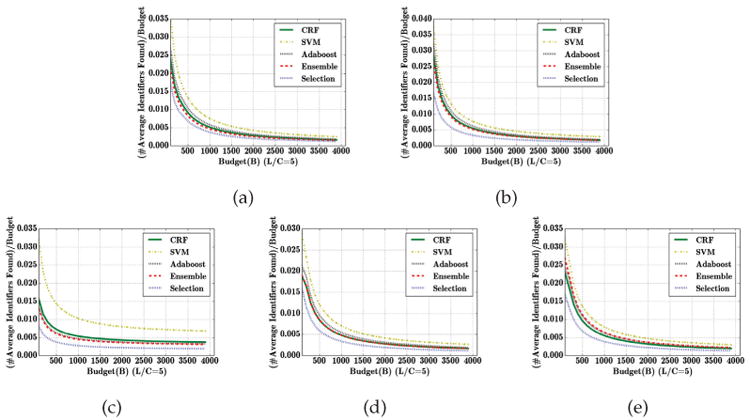
The ratio of the average number of sensitive identifiers found by the attacker and the adversarial budget, while the publisher applies classifiers CRF, SVM, AdaBoost, Ensemble, and Selection which allows the publisher to choose a learner with highest accuracy from {CRF, SVM, AdaBoost, Ensemble} for GS (L/C=5). (a) i2b2, (b) VUMC, (c) Enron, (d) Newsgroup, and (e) Reuters datasets.

**Fig. 7:**
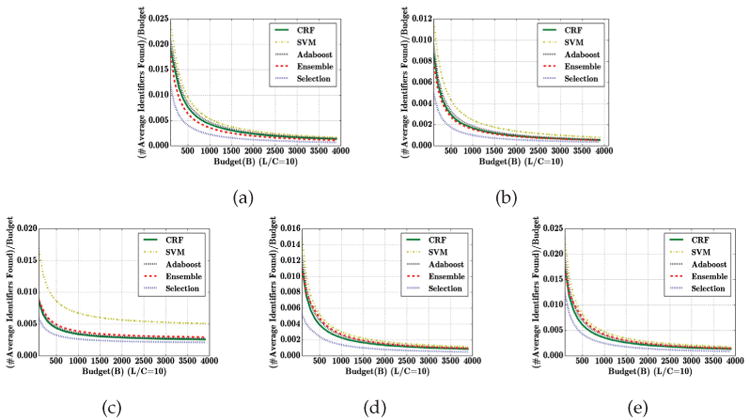
The ratio of the average number of sensitive identifiers found by the attacker and the adversarial budget, while the publisher applies classifiers CRF, SVM, Adaboost, Ensemble, and Selection which allows the publisher to choose a learner with highest accuracy from {CRF, SVM, Adaboost, Ensemble} for GS (L/C=10). (a) i2b2, (b) VUMC, (c) Enron, (d) Newsgroup, and (e) Reuters datasets.

Figures 6 and 7 compare these five options (the four single-classifier options, and the last, called “Selection”, where the most accurate of these classifiers is chosen in each iteration), evaluated when the adversary chooses the most accurate of these. Figure 6 considers *L/C* = 5 and Figure 7 presents results for *L/C* = 10. The overall observation is that increasing the space of classifiers to choose from is beneficial (indicated by the “Selection”, which chooses the best classifier of the collection leaving fewer uncovered identifiers), but the difference is relatively small. Moreover, the number of identifiers discovered by the attacker as a fraction of budget in all cases remains extremely small. Consequently, significant underestimation of the attacker's strength appears unlikely to make much impact. It is also revealing that the classifiers tend to perform similarly (except SVM, which is often substantially worse than the others), and better classifiers (such as CRF) tend to lead to better performance of GS.

### Number of Greedy Iterations

**Fig. 8:**
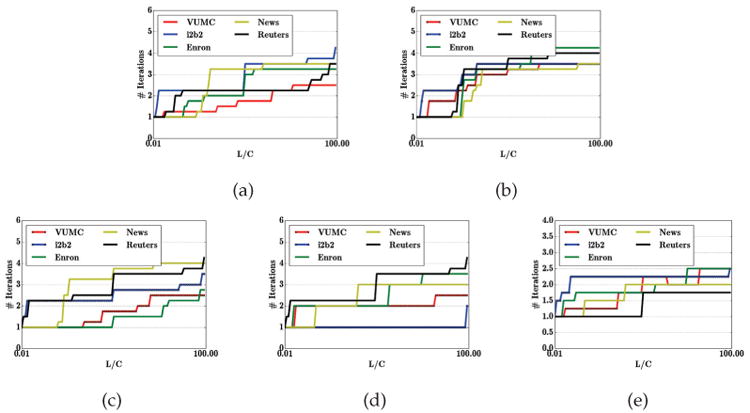
The number of iterations of GS for i2b2, VUMC, Enron, and Newsgroup datasets, where publisher chooses (a) CRF, (b) SVM, (c) Adaboost, (d) Ensemble, and (e) the best algorithm from {CRF, SVM, Adaboost, Ensemble}, respectively.

The final issue we consider is the number of iterations of GS (and, consequently, the number of classifiers it uses) for the different data sets. Here we evaluate the convergence rate for the GS algorithm when applying different baseline algorithms. It is clear that GS converges in a small number iterations regardless what underlying algorithm is used. Specifically, Figure 8 shows that for all five datasets (and for the entire range of *L/C* that we consider) the average number of iterations is less than 5, significantly better than our |*X*| bound! Our theoretical upper bound is, therefore, extremely pessimistic. Indeed, for some datasets, such as the VUMC EMR dataset, the average number of iterations is just above 2 - even when the loss from leaking sensitive information is quite high. In practice, it appears, the effectiveness of learning degrades quite rapidly, making it extremely difficult for attackers to obtain any residual re-identification value from published data.

## 6 Conclusion

Our ability to take full advantage of large amounts of unstructured data collected across a broad array of domains is limited by the sensitive information contained therein. This paper introduced a novel framework for sanitization of such data that relies upon 1) a principled threat model, 2) a very general class of publishing strategies, and 3) a greedy, yet effective, data publishing algorithm. The experimental evaluation shows that our algorithm is: a) substantially better than existing approaches for suppressing sensitive data, and b) retains most of the value of the data, suppressing less than 10% of information on all four data sets we considered in evaluation. In contrast, cost-sensitive variants of standard learning methods yield virtually no residual utility, suppressing most, if not all, of the data, when the loss associated with privacy risk is even moderately high. Since our adversarial model is deliberately extremely strong - far stronger, indeed, than is plausible - our results suggest feasibility for data sanitization at scale.

## Figures and Tables

**Table 1:** Table of Notations

*n*	≜	number of total instances
*H*	≜	hypothesis class of the publisher
*H*	≜	the subset of classifiers chosen by the publisher
*S*	≜	sensitive instances
*N*	≜	non-sensitive instances
*TP*(*h, P*)	≜	number of *true positives* by *h* on *P*
*TN*(*h, P*)	≜	number of *true negatives* by *h* on *P*
*FP*(*h, P*)	≜	number of *false positives* by *h* on *P*
*FN*(*h, P*)	≜	number of *false negatives* by *h* on *P*
*TP_A_*	≜	number of *true positives* obtained by attacker
*TN_A_*	≜	number of *true negatives* obtained by attacker
*FP_A_*	≜	number of *false positives* obtained by attacker
*FN_A_*	≜	number of *false negatives* obtained by attacker
*TP_D_*	≜	number of *true positives* obtained by defender
*TN_D_*	≜	number of *true negatives* obtained by defender
*FP_D_*	≜	number of *false positives* obtained by defender
*FN_D_*	≜	number of *false negatives* obtained by defender
*α*	≜	percent of identifiers in data
*h_A_*	≜	the attacker's classifier
*T*(*H*)	≜	loss function of data publisher for *H*

**Table 2:** Statistics of Datasets

Dataset	Words	Documents	Sensitive Instances
i2b2	386,736	664	6853
VUMC	226,455	600	5154
Enron	120,131	761	6084
Newsgroup	119,303	597	3525
Reuters	324,950	788	17050

## Footnotes

1 Typical ensemble learning algorithms will either focus on mistakes made in prior iterations (e.g., boosting), ignore the performance of other members of the ensemble (e.g., bagging), or use a fixed set of classifiers as inputs into a meta-classifier [61].

2 For simplicity, we assume that *m* is also the size of the residual test data that is ultimately released. Generalization of the results below is relatively direct.

## Appendix A

### Proof of Lemma 4

*Proof.* By Theorem 2, Algorithm 1 will terminate when $\frac{\hat{FP_{A}}}{\hat{TP_{A}}}\geq \frac{L}{C}$. Using, Equation 5 and our assumptions, we have

$$\frac{(1+\frac{1}{p})\hat{FP_{A}}}{m}\leq \frac{(1+\frac{1}{s})FP_{A}}{m}+\lambda (m,\delta )$$

with probability at least 1−*δ*. Consequently with probability at least 1−*δ*,

$$\frac{(1+\frac{1}{p})\hat{FP_{A}}}{TP_{A}\cdot \hat{TP_{A}}}\leq \frac{(1+\frac{1}{s})FP_{A}}{TP_{A}\hat{TP_{A}}}+\lambda (m,\delta )\frac{m}{\hat{TP_{A}}TP_{A}},$$

and, consequently

$$\frac{1+\frac{1}{p}}{TP_{A}}\cdot \frac{L}{C}\leq \frac{(1+\frac{1}{s})FP_{A}}{TP_{A}\hat{TP_{A}}}+\lambda (m,\delta )\frac{m}{\hat{TP_{A}}TP_{A}}.$$

Rearranging, we get

$$\begin{array}{c} \frac{FP_{A}}{TP_{A}}\geq \frac{(1+\frac{1}{p})\frac{L}{C}\cdot \frac{1}{TP_{A}}-\lambda (m,\delta )\cdot \frac{m}{\hat{TP_{A}}}\cdot \frac{1}{TP_{A}}}{(1+\frac{1}{s})\cdots \frac{1}{\hat{TP_{A}}}}\\ =\frac{1}{1+\frac{1}{s}}(\frac{L}{C}(1+\frac{1}{p})\cdot \frac{\hat{TP_{A}}}{m}\cdot \frac{m}{TP_{A}}-\lambda (m,\delta )\cdot \frac{N_{A}}{TP_{A}})\\ \geq (\frac{1}{1+\frac{1}{s}})((1+\frac{1}{p})\frac{\hat{TP_{A}}}{m}\frac{L}{C}-\lambda (m,\delta ))\frac{m}{TP_{A}}\\ \geq (\frac{1}{1+\frac{1}{s}})((1+\frac{1}{p})\cdot q\cdot \frac{L}{C}-\lambda (m,\delta ))\frac{1}{r} \end{array}$$

## Appendix B

### Proof of Theorem 5

*Proof.* Since

$$\begin{array}{c} \frac{FP_{A}}{TP_{A}}=\frac{TN_{D}-TN_{A}}{TP_{A}}\\ \geq (\frac{1}{1+\frac{1}{s}})((1+\frac{1}{p})\cdot q\cdot \frac{L}{C}-\lambda (m,\delta ))\frac{1}{r} \end{array}$$

$$\begin{array}{c} TP_{A}\leq r(1+\frac{1}{s})(\frac{1}{\left(1+\frac{1}{p})\cdot q\cdot \frac{L}{C}-\lambda (m,\delta \right)})(TN_{D}-TN_{A})\\ \leq r(1+\frac{1}{s})(\frac{1}{\left(1+\frac{1}{p})\cdot q\cdot \frac{L}{C}-\lambda (m,\delta \right)})\cdot TN_{0}\\ =r(1+\frac{1}{s})\frac{(1-\alpha )n}{\left(1+\frac{1}{p})\cdot q\cdot \frac{L}{C}-\lambda (m,\delta \right)}. \end{array}$$

## Appendix C

### Proof of Theorem 6

*Proof*.

$$U_{A}=L\cdot B\cdot \frac{TP_{A}+FN_{A}}{TP_{A}+FP_{A}+FN_{A}+TN_{A}}.$$

Based on the general adversarial model, the attacker can always choose the priority to guarantee *U_A*_* ≥ *U_A_* according to Lemma 2. Therefore, when *TP_A_TN_A_* ≥ *FP_A_FN_A_*, the attacker prioritizes the positives than negatives, so $U_{A*}=L\cdot (TP_{A}+\frac{FN_{A}}{FN_{A}+TN_{A}}(B-TP_{A}-FP_{A}))$. Therefore we have

$$\begin{array}{c} \frac{U_{A*}}{U_{A}}=1+\frac{TP_{A}TN_{A}-FP_{A}FN_{A}}{\left(TP_{A}+FP_{A})(TP_{A}+FN_{A}\right)}\\ =1+\frac{TN_{A}-\frac{FP_{A}}{TP_{A}}\cdot FN_{A}}{\left(1+\frac{FP_{A}}{TP_{A}})(TP_{A}+FN_{A}\right)}\\ \leq 1+\frac{TN_{A}-\frac{FP_{A}}{TP_{A}}}{1+\frac{FP_{A}}{TP_{A}}}\\ \leq 1+\frac{\left(1-\alpha )nr(1+\frac{1}{s})-((1+\frac{1}{p})q\frac{L}{C}-\lambda (\delta ,m)\right)}{r(1+\frac{1}{s})+(1+\frac{1}{p})q\frac{L}{C}-\lambda (\delta ,m)}\\ =\frac{\left((1-\alpha )n+1)r(1+\frac{1}{s}\right)}{r(1+\frac{1}{s})+(1+\frac{1}{p})q\frac{L}{C}-\lambda (\delta ,m)}. \end{array}$$
